# Various Strategies of Tendon Stem/Progenitor Cell Reprogramming for Tendon Regeneration

**DOI:** 10.3390/ijms252111745

**Published:** 2024-11-01

**Authors:** Sung Yong Ahn

**Affiliations:** Department of Physiology, Ajou University School of Medicine, Suwon 16499, Republic of Korea; davidahn@ajou.ac.kr or davidahn@aumc.ac.kr or ahnsungyong83@gmail.com; Tel.: +82-10-8502-4011

**Keywords:** tendon stem/progenitor cell, cell reprogramming, rotator cuff disease, extracellular vesicles, small molecules, regeneration

## Abstract

Rotator cuff tears (RCT) are the most common cause of shoulder pain among adults. “Rotator cuff” refers to the four muscles that cover the shoulder joint: supraspinatus, infraspinatus, subscapularis, and teres minor. These muscles help maintain the rotational movement and stability of the shoulder joint. RCT is a condition in which one or more of these four muscles become ruptured or damaged, causing pain in the arms and shoulders. RCT results from degenerative changes caused by chronic inflammation of the tendons and consequent tendon tissue defects. This phenomenon occurs because of the exhaustion of endogenous tendon stem cells. Tendon regeneration requires rejuvenation of these endogenous tendon stem/progenitor cells (TSPCs) prior to their growth phase. TSPCs exhibit clonogenicity, multipotency, and self-renewal properties; they express classical stem cell markers and genes associated with the tendon lineage. However, specific markers for TSPC are yet to be identified. In this review, we introduce novel TSPC markers and discuss various strategies for TSPC reprogramming. With further research, TSPC reprogramming technology could be adapted to treat age-related degenerative diseases, providing a new strategy for regenerative medicine.

## 1. Introduction

Shoulder pain is a common musculoskeletal complaint, affecting 16% of the general population and 21% of older people [1,2,3]. It is the most prevalent cause of musculoskeletal pain among individuals in their 40s and 50s—the age group where economic productivity is at its peak with a prevalence of 16.9%, followed by back pain (15.9%), and knee pain (10.7%) [4]. Several factors can cause shoulder pain, but the two main causes are frozen shoulder and rotator cuff disease; of these, rotator cuff disease accounts for approximately 50–70% of cases [1,2,3].

Tendons are fibrous soft tissues that serve as connective structures between muscles and bones [5]. They are primarily made up of well-organized type I collagen fibers and spindle-shaped cells, the majority of which are mature tenocytes. Tenocytes and tendon-derived stem/progenitor cells (TSPCs) are the fundamental cell types found in tendon tissues. Tenocytes are a particular type of fibroblast that account for approximately 95% of tendon tissue [6].

TSPCs are distinct cell types that are capable of self-renewal, differentiation into multiple cell lineages, and exhibit clonogenicity; they were initially identified in mouse patellar tendon tissue by Bi et al. [7]. TSPCs are a recently discovered cell type that have been identified in both human and rat tendons, particularly in areas with high levels of biglycan (Bgn) and fibromodulin (Fmod) in the extracellular matrix [7]. Subsequently, TSPCs have also been obtained from other areas, such as the patellar tendon, Achilles tendon, supraspinatus tendon, hamstring tendon, and other sites, in rats, rabbits, pigs, and humans [8,9,10]. Structurally, TSPCs are smaller than tenocytes, possess larger nuclei, and display a faster proliferation rate compared with tenocytes [10]. No specific markers for TSPC have yet been identified [11]. However, dissimilar to other stem cells, TSPCs express higher levels of collagen and tendon-related genes, such as scleraxis (Scx), tenomodulin (Tnmd), and tenascin-C (TNC). Several recent studies have reported the identification of novel TSPC markers. Tendon tissue homeostasis, repair, and regeneration are assumed to be critical functions of TSPCs [7,9,10], and a decline in the number or function of TSPCs is suggested to cause aging [12] or tendinopathy [13], often leading to tendon degeneration [14,15].

Previous research has shown that reduced activity of skeletal muscle stem cells (satellite cells) due to loss of Notch signaling impairs the regeneration of aged muscles [16,17]. Similarly, a decrease in liver progenitor cell proliferation due to the formation of a complex containing cEBP-α and the chromatin remodeling factor Brahma (Brm) inhibits the regenerative capacity of an aged liver [18]. A promising approach that could potentially overcome the challenges associated with cell transplantation is the use of endogenous stem/progenitor cells to regenerate tissues. This concept has recently gained attention as a feasible approach to advancing regenerative medicine. This review identifies novel markers of TSPC and suggests that overcoming TSPC depletion could be a direct strategy for achieving tendon regeneration. Therefore, this review summarizes several strategies for tendon regeneration through TSPC reprogramming.

## 2. Causes of Rotator Cuff Disease

The term ‘rotator cuff’ refers to four muscles that surround the shoulder joint: the supraspinatus, infraspinatus, subscapularis, and teres minor. These muscles play a role in maintaining the rotational movement and stability of the shoulder joint. Rotator cuff tear (RCT) is a condition in which one or more of these four muscles become ruptured or injured, causing pain in the arm and shoulder. The cause of rotator cuff disease is controversial and not clearly understood. Tendon degeneration is thought to be one of the most important causes of rotator cuff disease [19]. According to a recent study, the pro-inflammatory environment plays an important role in causing degenerative changes in the tendon [20,21]. A well-known cause of rotator cuff disease is chronic inflammation of the tendon tissues and subsequent tendon tissue defects. Age-related degenerative changes are also significant contributors. A recent study has defined stem cell exhaustion and decreased regenerative capacity as hallmarks of aging [22]. Stem cell exhaustion refers to a decline in the number of stem cells and their renewal capacity. Without a stable population of proliferating stem cells, tissues and organs lose their ability to recover from damage, ultimately resulting in organ failure. The depletion of stem cells may be primarily caused by specific forms of age-related dysfunction, including diminished self-renewal abilities and activation of cellular senescence mechanisms. In 2017, Howell et al. [23] showed that for tendon regeneration, instead of transferring exogenous cells to the damaged region, endogenous stem cells present within the tendon tissue must be moved to the damaged area, proliferated, and then induced to differentiate into tendons. Thus, among the various factors causing rotator cuff disease, the exhaustion of endogenous tendon stem cells is the root cause. To treat such degenerative diseases, it is important to restore the number and function of endogenous stem cells in the tissue. In other words, a biological strategy targeting the rejuvenation of endogenous stem cells is necessary.

## 3. Identification and Characterization of Tenocytes and TSPCs

Regenerating normal structure and function in injured tendons presents a significant challenge in orthopedics and regenerative medicine. Tendons are organized in a hierarchical structure comprising fascicles, fibers, and fibrils and are mostly composed of collagen molecules. Despite being predominantly made of tenocytes, tendons contain a pool of stem and progenitor cells [24]. The utilization of TSPCs in regenerative medicine is a promising strategy for addressing tendon injuries, as they can potentially serve as a potent cell-based therapy for facilitating tendon healing and regeneration. Prior research has shown that tendon aging is closely related to changes in TSPC function [25,26]. In 2007, Bi et al. [7] reported the isolation of a cell population (TSPCs) from both humans and mice, which exhibited characteristics typical of stem cells, such as multipotency, clonogenicity, and self-renewal properties. Despite multiple investigations, the identification process for TSPCs remains imprecise and lacks specific markers [27]. This is one of the reasons why TSPC research remains in the in vitro stage. The isolation of tenocytes and TSPCs follow similar cell extraction protocols. Additionally, the morphology of TSPCs is similar to that of tenocytes, and there is insufficient information to compare and identify them, making the in vitro study of tendons difficult. Although most studies do not distinguish between these two types of cells, it is still important because endogenous TSPCs can facilitate tendon regeneration in musculoskeletal conditions such as RCT [28,29,30]. TSPCs are positive for certain common stem cell markers that are also present on the surface of other mesenchymal stem cells (MSCs), including Sca-1, CD44, CD90, CD90.1, CD105, CD146, Stro-1, nucleostemin, Oct-4, and SSEA-1, but not CD18, CD31, CD34, CD45, CD106, CD117, CD144, or Flk-1 [31]. However, no molecular markers have been identified that allow us to distinguish between TSPCs and tenocytes. The precise location of TSPCs in tendons also remains unclear. Recently, two different regions of stem/progenitor cells have been observed within the tendon, specifically in the outer tendon sheet (TSPC type I) and within the tendon proper (TSPC type II) [32,33]. Additionally, cells that concurrently expressed genes associated with tendon and pericyte markers were found in the perivascular region of tendon tissues. This observation suggests that the perivascular niche may serve as a reservoir for another distinct population of local stem cells or progenitor cells [34]. Comparison between subpopulations of TSPCs revealed that peritenon-derived cells (TSPC type I) had increased vascular and pericyte markers, while tendon proper-derived cells (TSPC type II) were more proliferative and exhibited higher levels of Scx and Tnmd [32]. In contrast to MSCs of other origins, TSPCs express the tendon-related genes Scx, Tnmd [35], cartilage oligomeric matrix protein (Comp), and TNC [7,9,10,34,36,37]. Therefore, there is a critical need for in vitro and in vivo studies to determine the role and localization of TSPCs in the tendons.

## 4. Novel TSPC Markers

As previously mentioned, the absence of novel TSPC markers is the primary obstacle to advancing TSPC research. A previous study reported CD146 as one of the markers that identifies TSPCs. They showed that TSPCs exist within a unique niche in the extracellular matrix and identified biglycan (Bgn) and fibromodulin (Fmod) as two important components of this niche [7]. Another study showed that CD146+ cells from freshly isolated rat patellar tendons accounted for approximately 0.8% of the total mononucleated cell population. However, CD146+ cells sorted by fluorescence-activated cell sorting (FACS) analysis were positive for expression of other TSPC markers, including CD44 and CD90 [38]; both these markers were also highly expressed in fetal MSCs [39]. The tubulin polymerization-promoting protein family member 3 (Tppp3) is a distinct marker for the development of the musculoskeletal system. Tppp3 is expressed in cells of connective tissues, such as the tendon sheath, epitenon, and paratenon surrounding the developing tendon [40]. Studies conducted using single-cell transcriptomics have reported that the Tppp3+ cell population might be tendon stem cells, which also expressed platelet-derived growth factor receptor alpha (PDGFRA). These Tppp3+ PDGFRA+ cells not only contribute to tendon regeneration but are also novel markers of tendon stem cells [41].

Another study based on single-cell analysis revealed that nestin+ TSPCs had strong tenogenic potential [42]. This study showed that nestin was involved in specific stages of limb and tendon development in NES-green fluorescent protein (GFP) transgenic mice. Depletion of nestin expression after shRNA treatment in TSPCs led to a decrease in clonogenic capability and diminished tenogenic potential both in vitro and in vivo. This indicates that nestin is essential for the preservation of the tendon-lineage phenotype in TSPCs [42]. Surface marker analysis of TSPCs showed Sca1+ (stem cell marker), CD90+ and CD44+ (fibroblast markers), CD18^–^ (leukocyte marker), CD34^–^ (hematopoietic and vascular marker), and CD133^–^ (perivascular marker). The expression levels of Tnmd and Scx were higher in TSPCs, suggesting that these cells were enriched in stem/progenitor cells of tendon origin [32]. Tnmd is widely recognized as the most prominent mature marker for tendon and ligament progenitor cells. A recent study showed that Tnmd is necessary to prevent adipocyte accumulation and fibrovascular scar formation during early tendon healing. This suggests that Tnmd is essential for tendon maturation and healing and has a critical impact on resident TSPCs [43].

## 5. Various Strategies for TSPC Reprogramming

### 5.1. Part A: Transcription Factors

Induced pluripotent stem cell (iPSC) reprogramming was originally performed using a set of transcription factors, including Oct4, Sox2, Klf4, and c-Myc (OSKM) [44]. Although iPSCs have great potential, they pose not only technical concerns (such as efficiency) but also biological risks, such as cancer formation and stability issues due to gene insertion. Transcription factor-induced reprogramming can have several notable side effects both in vitro and in vivo. A recent study reported that inducing OSKM for a relatively short period of seven days can lead to cancer [45]. Reprogramming often causes extensive damage to the cells, leading to cellular aging. Moreover, one of the major risks of transcription factor-induced reprogramming is the potential formation of teratomas that arise from pluripotent stem cells [46]. Reprogramming can also lead to epigenetic changes that are not completely reversible. For example, certain studies have shown instability in the X chromosome of human induced pluripotent stem cells (iPSCs), which can affect the therapeutic potential of these cells [47]. Many transcription factors are resistant to reprogramming, making the process inefficient and affecting only a small subset of cells. The process also requires a long latency, which complicates its potential therapeutic applications [48].

Several researchers have attempted to convert tissue-differentiated cells into induced tissue-specific stem cells (iTSCs) or partially/transiently reprogram them instead of iPSCs [49,50]. Therefore, efforts have been made to avoid or reduce the use of some of these transcription factors, and several studies have attempted reprogramming using only one or two of Oct4, Sox2, and Klf4 [51,52,53].

### 5.2. Part B: Small Molecules

Alternative methods have been explored to reduce the risk of gene transduction, including the use of small molecules to promote reprogramming [54]. Several chemicals have recently been reported to enhance reprogramming efficiencies or replace defined reprogramming factors, such as OSKM [55]. In 2011, Li et al. [56] developed a small molecule combination, VC6T [Valpronic acid (VPA), CHIR99021 (CHIR), 616452 (6), Tranylcypromine (T)], which enables reprogramming using only a single gene, Oct4. In 2013, Hou et al. [57] confirmed the efficacy of a VC6TFZ small-molecule cocktail in mouse embryonic fibroblast cells by combining VC6T with F, forskolin (FSK) and Z, 3-deazaneplanocin (DZNep). In 2023, Yang et al. [58] investigated the optimal combination of small molecules in humans (C6NYSA: CHIR, 616452, TTNPB (N), Y27632 (Y), Smoothened agonist (SAG), and ABT869 (A)) and mice (VC6TF: VPA, CHIR, 616452, T, and FSK) cells. Researchers have used different combinations of small molecules in humans and mice because of differences in differentiation between humans and mice. Numerous chemicals have been identified to affect chromatin modifications and signal transduction pathways (Table 1). Researchers have recently succeeded in converting somatic cells into chemically-induced pluripotent stem cells (CiPSCs) by using a combination of reprogramming boosters, suggesting the possibility of rejuvenation through partial reprogramming [57,59]. A combination of six small-molecule compounds was found to reverse the age of the transcriptome in both humans and mice with no loss of cell identity [58]. The unique feature here is that a combination of the six small-molecule compounds differed for humans and mice, which may be due to the differences in differentiation between the two organisms. Therefore, tendon regeneration through TSPC reprogramming can be achieved not only through genetic manipulations but also through chemical means.

### 5.3. Part C: Extracellular Vesicles

**Figure 1:**
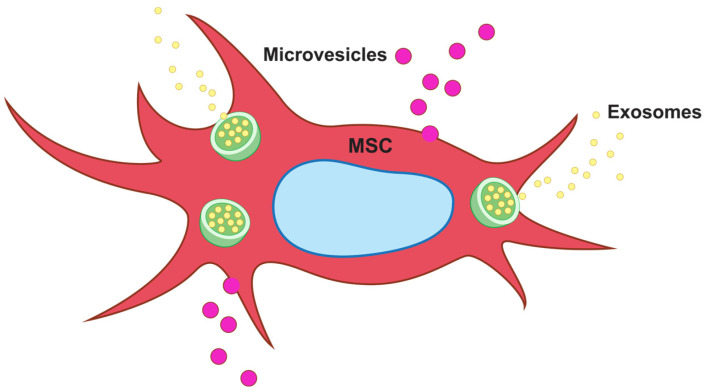
Schematic representation of mesenchymal stem cell (MSC)-derived extracellular vesicles (EVs). EVs include apoptotic bodies (>1000 nm), microvesicles (MVs: 100–1000 nm), and exosomes (30–150 nm). Exosomes are released by a variety of cell types, such as T cells, B cells, cancer cells, and stem cells, and transport proteins, lipids, and genetic materials, including DNA and RNA, to nearby or remote cells.

Extracellular vesicles (EVs) are membrane-enclosed vesicles secreted by cells that function by transporting bioactive molecules to target cells. EVs include apoptotic bodies (>1,000 nm), microvesicles (MVs: 100–1,000 nm), and exosomes (30–150 nm). Several cell types secrete exosomes, including T cells, B cells, cancer cells, and stem cells (Figure 1). Exosomes are messenger particles that are naturally released from the cells, which carry proteins, lipids, and genetic materials such as DNA and RNA to neighboring or distant cells. Exosomes can reprogram recipient cells based on their bioactive compounds [69]. In 2005, Conboy et al. [70] showed the rejuvenation of aged stem cells due to heterochronic parabiosis and confirmed that the rejuvenation of stem cells was caused by secretomes in young tissues. Recent studies have revealed the possibility of rejuvenating aged MSCs using exosomes derived from iPSCs and embryonic stem cells (ESs) [71,72]. In particular, Yu et al. [73] reported that exosomes from bone marrow-derived mesenchymal stem cells (BM-MSCs) promote the proliferation and migration of CD146+ endogenous stem cells in the tendon. The effect of exosomes on the rejuvenation of endogenous stem cells is different from the anti-inflammatory and immunomodulatory effects of existing exosomes on differentiated cells. Thus, exosomes can be used as tools for fundamental tissue regeneration.

### 5.4. Part D: Fetal MSCs

In 2020, Khanh et al. [74] showed that treatment with elderly stem cell-derived exosomes (eEVs) did not alter aged stem cells, but treatment with infant stem cell-derived exosomes (iEVs) significantly reduced aging. Senescence changes the composition of the stem cell secretome, which is assumed to cause stem cell exhaustion—a key cause of senescence-related degenerative diseases. In particular, in the case of human umbilical cord-derived mesenchymal stem cells (hUC-MSCs), the possibility of treating myocardial infarction through the rejuvenation of aged BM-MSCs has been reported [75]; therefore, fetal MSC-derived exosomes can be used for the rejuvenation of senescent stem cells.

## 6. Discussion

A rotator cuff tear is a musculoskeletal condition with the fastest increasing incidence and morbidity rates. In the early stages of rotator cuff disease, conservative treatments such as rest, medication, physical therapy, and steroid injection are the primary treatment strategies; however, as this treatment does not address the cause, approximately 41% of patients still complain of symptoms one year after conservative treatment, and in some cases, this progresses to RCT [76]. Rotator cuff disease mainly occurs because of chronic inflammation and the resulting tissue defects due to the exhaustion of endogenous stem cells in tissues. The mechanisms underlying the occurrence of musculoskeletal degenerative disorders are as follows [77]. (1) Exhaustion of endogenous stem cells because of the accumulation of internal and environmental factors. (2) Changes in cell–matrix and cell–cell communication accompanying inflammatory responses. (3) The occurrence of degenerative disorders such as tissue defects and delayed healing due to continued chronic inflammation. Stem cell exhaustion refers to a decline in the function and number of stem cells in a tissue. Age-related degeneration can be summarized into three stages. Stem cell senescence occurs with advancing age and results in a concomitant decline in stem cell functionality. This functional decline alters exosome-mediated extracellular communication between stem cells and their progeny, thereby contributing to diverse disease etiologies [77]. Stem cell exhaustion occurs in various organs and contributes to both aging and disease progression. Hematopoietic stem cells (HSCs) are responsible for blood cell formation and face exhaustion because of chronic stressors such as infections, chemotherapy, and aging. This leads to diminished regenerative capacity and impaired blood cell production, contributing to conditions such as leukemia and anemia [78,79]. T cell exhaustion occurs primarily in organs affected by chronic infections or cancers, such as the lungs and lymphoid tissues. This leads to impaired immune responses and a reduced ability to eliminate tumors or persistent infections [80]. Mesenchymal stem cells (MSCs) found in organs such as the kidneys, liver, and lungs are exhausted owing to chronic diseases or injury. This affects the regenerative ability of these organs and contributes to fibrosis and reduced organ function [81]. Muscle stem cells (satellite cells) and skin stem cells experience exhaustion with aging, leading to reduced tissue regeneration, contributing to frailty and skin thinning. Autophagy plays a key role in the prevention of stem cell exhaustion in these tissues [82]. Stem cell exhaustion is a critical factor in aging and diseases, affecting multiple organs, such as the bone marrow, immune system, muscles, and skin. Studies on neonatal and adult rats have proven that for tendon regeneration in adults, instead of transferring extrinsic cells to the damaged area, intrinsic stem cells existing within the tendon must be moved to the damaged area, proliferated, and then induced to initiate tendon differentiation [23]. In an interesting study using ^14^C bomb pulse curves in 2013, Heinemeier et al. [83,84] reported that the core collagen that forms the Achilles tendon is formed from birth until the growth period of 13–17 years and is not reformed thereafter. Therefore, tendon regeneration requires the rejuvenation of endogenous stem cells prior to the growth phase. Recently, there has been the possibility of stem cell rejuvenation through short-term expression of defined factors, such as Oct4, Sox2, Klf4, and c-Myc (OSKM), or partial reprogramming to rejuvenate differentiated tissue cells into induced tissue-specific stem cells (iTSCs) [49,50]. Many studies have used various reprogramming boosters and defined factors to promote rejuvenation [55]. In addition, they succeeded in converting mouse somatic cells into chemically induced pluripotent stem cells (CiPSCs) using a combination of reprogramming boosters [57,59]. In a study on the rejuvenation of aged stem cells, it was reported that the rejuvenation caused by heterochronic parabiosis is caused by secretomes produced in young tissues [70]. In a recent study, the possibility of rejuvenation of aged MSCs by induced pluripotent stem cells (iPSCs) and embryonic stem cell (ES)-derived extracellular vesicles (EVs) was demonstrated [71,72]. These results suggest that exosomes have mechanisms that are different from the existing anti-inflammatory and immunomodulatory abilities and can be used as a tool for more fundamental tissue regeneration. Senescence changes the composition of the stem cell secretome and causes a decline in the number and function of stem cells, which is a key cause of age-related degenerative diseases. In the case of stem cells collected from patients with chronic diseases, various functions, such as proliferation and differentiation, were found to be poor [85]. Furthermore, in 2020, Weiss et al. [86] reported that the rejuvenation effect of EVs generated from adipose-derived stem cells (ASCs) in patients with chronic diseases, such as metabolic syndrome, was reduced compared with that in healthy individuals. Therefore, the rejuvenation of aged stem cells requires exosomes derived from fetal MSCs rather than adult MSCs.

## 7. Conclusions

**Figure 2:**
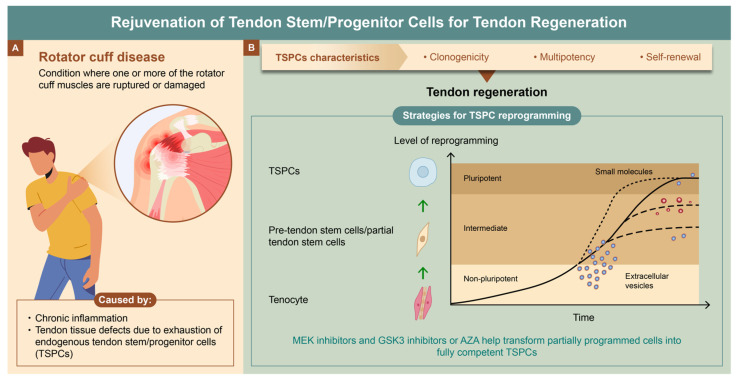
Rejuvenation of tendon stem/progenitor cells (TSPCs) for tendon regeneration. (**A**) Causes of rotator cuff disease. Chronic inflammation and tendon tissue defects due to exhaustion of TSPCs. (**B**) Strategies for TSPC reprogramming. Tendon regeneration through the rejuvenation of TSPCs using a combination of extracellular vesicles derived from fetal mesenchymal stem cells (MSCs) and small molecules.

In conclusion, it was suggested the rejuvenation of endogenous TSPCs is necessary for the treatment of rotator cuff disease. To rejuvenate aged endogenous stem cells to the growth stage, a combination of extracellular vesicles derived from fetal MSCs and small molecules is required (Figure 2). The exhaustion of endogenous stem cells is the most fundamental cause of age-related degenerative diseases in the human body. In the future, reprogramming technology of endogenous stem cells can be expanded to treat various diseases and injuries as a new strategy for regenerative medicine.

## Figures and Tables

**Table 1:** Chemicals used to enhance reprogramming efficiency or replace essential reprogramming factors.

No.	Author /Year/Journal	Title	Chemical Used	Details	Yamanaka Factor Used	Species and Cell Type	Inference	Ref.
1	Shi et al./2008a/Cell Stem Cell	A combined chemical and genetic approach for the generation of induced pluripotent stem cells	BIX01294	G9a histone methyltransferase inhibitor	OK	mouse neural progenitor cells (mNPCs)	OK+BIX01294 enhances efficiency ~1.5 times more than OSKM and ~8 times more than OK; BIX01294 is able to replace S and M.	[60]
			BIX01294	G9a histone methyltransferase inhibitor	KSM	fetal neural progenitor cells (fNPCs)	BIX01294 is able to replace O in NPC reprogramming but with extremely low efficiency.	
2	Shi et al./2008b/Cell Stem Cell	Induction of pluripotent stem cells from mouse embryonic fibroblasts by Oct4 and Klf4 with small-molecule compounds	BIX01294	G9a histone methyltransferase inhibitor	OK	mouse embryonic fibroblasts (MEFs)	OK+BIX01294 enhances efficiency ~5 times more than OK and is able to replace S.	[61]
			BayK8644	L-type calcium channel agonist	OK	MEFs	OK+BIX01294+BayK8644 enhances efficiency ~15 times more than OK.	
			RG108	DNA methyltransferase (DNMT) inhibitor	OK	MEFs	OK+BIX01294+RG108 enhances reprogramming efficiency ~30 times more than OK.	
3	Mikkelsen et al./2008/Nature	Dissecting direct reprogramming through integrative genomic analysis	AZA	DNMT inhibitor	OSKM	MEFs	AZA treatment during days 8–10 resulted in a ~4-fold increase in efficiency compared with untreated controls.	[62]
4	Huangfu et al./2008a/Nature Biotechnology	Induction of pluripotent stem cells by defined factors is greatly improved by small-molecule compounds	VPA	histone deacetylase (HDAC) inhibitor	OSKM	MEFs	More than 100-fold increase in efficiency with OSKM.	[63]
			AZA	DNMT inhibitor	OSK	MEFs	~3-fold increase in efficiency with OSK.	
			VPA	HDAC inhibitor	OSK	MEFs	~50-fold increase in efficiency with OSK.	
			Dexamethasone (dex)	synthetic glucocorticoid	OSKM	MEFs	Improved the effect of 5′-azaC by 2.6-fold when used in combination, even though dex alone had no significant effect.	
			TSA	HDAC inhibitor	OSKM	MEFs	~15-fold increase in efficiency with OSKM.	
			SAHA	HDAC inhibitor	OSKM	MEFs	~2-fold increase in efficiency with OSKM.	
5	Huangfu et al./2008b/Nature Biotechnology	Induction of pluripotent stem cells from primary human fibroblasts with only Oct4 and Sox2	VPA	HDAC inhibitor	OSK	human fibroblasts	10- to 20-fold increase compared with OSK (reprogramming efficiency 1.1%).	[64]
			VPA	HDAC inhibitor	OS	human fibroblasts	VPA is able to replace K and M (reprogramming efficiency 0.001%).	
6	Silva et al./2008/PLOS Biology	Promotion of reprogramming to ground state pluripotency by signal inhibition	PD0325901 + CHIR99021 (2i)	inhibitors of MEK and GSK3, respectively	OK	MEFs	Together with LIF, it promotes ground state pluripotency in OK pre-iPSCs	[65]
7	Li W et al./2009/Cell Stem Cell	Generation of rat and human-induced pluripotent stem cells by combining genetic reprogramming and chemical inhibitors	PD0325901 + CHIR99021 (2i) + A-83-01	Inhibitors of MEK, GSK3, and TGF-b1(ALK5), respectively	OSK	rat liver epithelial cells	Together with LIF and 2i, they generate mESC-like rat iPSCs	[66]
			PD0325901 + CHIR99021 (2i) + A-83-01	inhibitor of MEK, GSK3, and TGF-b1(ALK5) respectively	OSK	human fibroblasts	Together with LIF and 2i, they generate mESC-like human iPSCs	
8	Li et al./2011/Cell Research	Generation of iPSCs from mouse fibroblasts with a single gene, Oct4, and small molecules	VC6T	VPA, CHIR99021, 616452, tranylcypromine	O	mouse fibroblasts	A specific chemical combination that is sufficient to permit reprogramming from mouse embryonic and adult fibroblasts in the presence of a single transcription factor (Oct4) within 20 days, replacing Sox2, Klf4, and c-Myc.	[56]
9	Hou et al./2013/Science	Pluripotent stem cells induced from mouse somatic cells by small-molecule compounds	VC6TF	VPA, CHIR99021, 616452, tranylcypromine, forskolin	None	Oct4 promoter-driven GFP expression (OG)-MEFs	A GFP-positive cluster was generated using VC6TF on day 20 (D20) after chemical treatment. The expression of two pluripotency-related genes, Sall4 and Sox2, and the expression of several extraembryonic endoderm (XEN) markers, Gata4, Gata6, and Sox17 were significantly induced by VC6TF.	[57]
			VC6TFZ	VPA, CHIR99021, 616452, tranylcypromine, forskolin, DZNep	None	OG-MEFs	Morphology of a compact, epithelioid, GFP-positive colony on day 32 (D32) after treatment	
			VC6TFZ with 2i-medium	VC6TFZ + 2i-medium	None	OG-MEFs	2i-competent, ESC-like, and GFP-positive cells obtained as chemically induced pluripotent stem cells (CiPSCs).	
10	Zhao et al./2015/Cell	A XEN-like state bridges somatic cells to pluripotency during chemical reprogramming	VC6TFZASD with N2B27-2iL	VPA, CHIR99021, 616452, tranylcypromine, forskolin, DZNep, AM580, SGC0946, 5-aza-dC + N2B27-2i medium + LIF	None	MEFs	The XEN-like state allows us to identify small-molecule boosters and establish a robust chemical reprogramming system with a yield ~1000-fold greater than that of the previously reported protocol.	[67]
11	Li X et al./2017/Cell Stem Cell	Direct reprogramming of fibroblasts via achemically induced XEN-like state	VC6TFAE	VPA, TD114-2/CHIR99021, 616452, tranylcypromine, forskolin, AM580, EPZ004777	None	MEFs, mouse postnatal fibroblasts (NBFs), and mouse adult lung fibroblasts (MAFs)	Functional neurons and hepatocytes can be induced from fibroblasts via a chemically induced and highly expandable XEN-like state, bypassing the pluripotent stage.Chemical induction increases the expression of XEN master genes (Gata4, Sall4, Sox17, and Gata6).	[59]
12	Guan et al./2022/Nature	Chemical reprogramming of human somatic cells to pluripotent stem cells	C6NYSA	CHIR99021, 616452, TTNPB, Y27632, SAG, ABT869	None	human embryonic fibroblasts (HEFs)	A cocktail of small molecules (CHIR99021, 616452, and TTNPB) converts human fibroblasts into epithelial-like cells. Additional small molecules (Y27632, ABT869, and SAG) further promoted the formation of epithelial-like cells.	[68]
13	Yang et al./2023/Aging	Chemically induced reprogramming to reverse cellular aging	VC6TF	VPA, CHIR99021, 616452, tranylcypromine, forskolin	None	mouse fibroblasts	Rejuvenation through age reversal can be achieved not only genetically but also chemically.Within a week, a cocktail of six chemicals succeeded in restoring the whole-genome transcriptional profile characteristic of youth and reversed transcriptional age without compromising cellular identity.	[58]
			C6NYSA	CHIR99021, 616452, TTNPB, Y27632, SAG, ABT869	None	human fibroblasts		

**Abbreviations:** MEFs, mouse embryonic fibroblasts; HEFs, human embryonic fibroblasts; LIF, leukemia inhibitory factor; mESC, mouse embryonic stem cell; GFP, green fluorescent protein; OG, Oct4 promoter-driven GFP expression; XEN, extraembryonic endoderm; DNMT, DNA methyltransferase; HDAC, histone deacetylase; V, valpronic acid (VPA); C, a GSK3-β inhibitor (CHIR99021); 6, a TGF-β inhibitor (616452); T, LSD1 inhibitor (Tranylcypromine); F, Forskolin (FSK); Z, SAH hydrolase inhibitor (3-deazaneplanocin, DZNep); 2i, GSK and MAPK inhibition; N, Retinoic Acid Receptor (RAR) Agonist (TTNPB); A, an RAR agonist (AM580); S, a DOT1L inhibitor (SGC0946); D, 5-aza-dC; S, Smoothened Agonist (SAG) HCI; A, ABT869 (Linifanib, RTK inhibitor); TD114-2, a preferable GSK3-β inhibitor; E, a DOT1L inhibitor (EPZ004777).

## Data Availability

No new data were created or analyzed in this study. Data sharing is not applicable to this article.

## References

[1] Urwin M., Symmons D., Allison T., Brammah T., Busby H., Roxby M., Simmons A., Williams G. (1998). Estimating the burden of musculoskeletal disorders in the community: The comparative prevalence of symptoms at different anatomical sites, and the relation to social deprivation. Ann. Rheum. Dis..

[2] Chard M.D., Hazleman B.L. (1987). Shoulder disorders in the elderly (a hospital study). Ann. Rheum. Dis..

[3] Chard M.D., Hazleman R., Hazleman B.L., King R.H., Reiss B.B. (1991). Shoulder disorders in the elderly: A community survey. Arthritis Rheum..

[4] Choi K., Park J.H., Cheong H.K. (2013). Prevalence of musculoskeletal symptoms related with activities of daily living and contributing factors in Korean adults. J. Prev. Med. Public Health.

[5] Thorpe C.T., Screen H.R. (2016). Tendon structure and composition. Adv. Exp. Med. Biol..

[6] Kannus P. (2000). Structure of the tendon connective tissue. Scand. J. Med. Sci. Sports.

[7] Bi Y., Ehirchiou D., Kilts T.M., Inkson C.A., Embree M.C., Sonoyama W., Li L., Leet A.I., Seo B.M., Zhang L. (2007). Identification of tendon stem/progenitor cells and the role of the extracellular matrix in their niche. Nat. Med..

[8] Yang J., Zhao Q., Wang K., Ma C., Liu H., Liu Y., Guan W. (2018). Isolation, culture and biological characteristics of multipotent porcine tendon-derived stem cells. Int. J. Mol. Med..

[9] Rui Y.F., Lui P.P., Li G., Fu S.C., Lee Y.W., Chan K.M. (2010). Isolation and characterization of multipotent rat tendon-derived stem cells. Tissue Eng. Part A.

[10] Zhang J., Wang J.H. (2010). Characterization of differential properties of rabbit tendon stem cells and tenocytes. BMC Musculoskelet. Disord..

[11] Dominici M., Le Blanc K., Mueller I., Slaper-Cortenbach I., Marini F., Krause D., Deans R., Keating A., Prockop D., Horwitz E. (2006). Minimal criteria for defining multipotent mesenchymal stromal cells. The International Society for Cellular Therapy position statement. Cytotherapy.

[12] Zhou Z., Akinbiyi T., Xu L., Ramcharan M., Leong D.J., Ros S.J., Colvin A.C., Schaffler M.B., Majeska R.J., Flatow E.L. (2010). Tendon-derived stem/progenitor cell aging: Defective self-renewal and altered fate. Aging Cell.

[13] Rui Y.F., Lui P.P., Wong Y.M., Tan Q., Chan K.M. (2013). Altered fate of tendon-derived stem cells isolated from a failed tendon-healing animal model of tendinopathy. Stem Cells Dev..

[14] Dunkman A.A., Buckley M.R., Mienaltowski M.J., Adams S.M., Thomas S.J., Satchell L., Kumar A., Pathmanathan L., Beason D.P., Iozzo R.V. (2013). Decorin expression is important for age-related changes in tendon structure and mechanical properties. Matrix Biol..

[15] Thornton G.M., Lemmex D.B., Ono Y., Beach C.J., Reno C.R., Hart D.A., Lo I.K. (2015). Aging affects mechanical properties and lubricin/PRG4 gene expression in normal ligaments. J. Biomech..

[16] Conboy I.M., Conboy M.J., Smythe G.M., Rando T.A. (2003). Notch-mediated restoration of regenerative potential to aged muscle. Science.

[17] Conboy I.M., Rando T.A. (2002). The regulation of Notch signaling controls satellite cell activation and cell fate determination in postnatal myogenesis. Dev. Cell.

[18] Iakova P., Awad S.S., Timchenko N.A. (2003). Aging reduces proliferative capacities of liver by switching pathways of EBPα growth arrest. Cell.

[19] Sano H., Ishii H., Yeadon A., Backman D.S., Brunet J.A., Uhthoff H.K. (1997). Degeneration at the insertion weakens the tensile strength of the supraspinatus tendon: A comparative mechanical and histologic study of the bone-tendon complex. J. Orthop. Res..

[20] Dakin S.G., Martinez F.O., Yapp C., Wells G., Oppermann U., Dean B.J., Smith R.D., Wheway K., Watkins B., Roche L. (2015). Inflammation activation and resolution in human tendon disease. Sci. Transl. Med..

[21] Abraham A.C., Shah S.A., Thomopoulos S. (2017). Targeting inflammation in rotator cuff tendon degeneration and repair. Tech. Shoulder Elb. Surg..

[22] Camernik K., Mihelic A., Mihalic R., Haring G., Herman S., Marolt Presen D., Janez A., Trebse R., Marc J., Zupan J. (2020). Comprehensive analysis of skeletal muscle- and bone-derived mesenchymal stem/stromal cells in patients with osteoarthritis and femoral neck fracture. Stem Cell. Res. Ther..

[23] Howell K., Chien C., Bell R., Laudier D., Tufa S.F., Keene D.R., Andarawis-Puri N., Huang A.H. (2017). Novel model of tendon regeneration reveals distinct cell mechanisms underlying regenerative and fibrotic tendon healing. Sci. Rep..

[24] Schneider M., Angele P., Jarvinen T.A.H., Docheva D. (2018). Rescue plan for Achilles: Therapeutics steering the fate and functions of stem cells in tendon wound healing. Adv. Drug Deliv. Rev..

[25] Kohler J., Popov C., Klotz B., Alberton P., Prall W.C., Haasters F., Muller-Deubert S., Ebert R., Klein-Hitpass L., Jakob F. (2013). Uncovering the cellular and molecular changes in tendon stem/progenitor cells attributed to tendon aging and degeneration. Aging Cell.

[26] Li Y., Dai G., Shi L., Lin Y., Chen M., Li G., Rui Y. (2019). The potential roles of tendon stem/progenitor cells in tendon aging. Curr. Stem Cell Res. Ther..

[27] Walia B., Huang A.H. (2019). Tendon stem progenitor cells: Understanding the biology to inform therapeutic strategies for tendon repair. J. Orthop. Res..

[28] Cao Y., Liu Y., Liu W., Shan Q., Buonocore S.D., Cui L. (2002). Bridging tendon defects using autologous tenocyte engineered tendon in a hen model. Plast. Reconstr. Surg..

[29] Wang B., Liu W., Zhang Y., Jiang Y., Zhang W.J., Zhou G., Cui L., Cao Y. (2008). Engineering of extensor tendon complex by an ex vivo approach. Biomaterials.

[30] Chen B., Wang B., Zhang W.J., Zhou G., Cao Y., Liu W. (2012). In vivo tendon engineering with skeletal muscle derived cells in a mouse model. Biomaterials.

[31] Lui P.P., Chan K.M. (2011). Tendon-derived stem cells (TDSCs): From basic science to potential roles in tendon pathology and tissue engineering applications. Stem Cell Rev. Rep..

[32] Mienaltowski M.J., Adams S.M., Birk D.E. (2013). Regional differences in stem cell/progenitor cell populations from the mouse achilles tendon. Tissue Eng. Part A.

[33] Docheva D., Muller S.A., Majewski M., Evans C.H. (2015). Biologics for tendon repair. Adv. Drug Deliv. Rev..

[34] Tempfer H., Wagner A., Gehwolf R., Lehner C., Tauber M., Resch H., Bauer H.C. (2009). Perivascular cells of the supraspinatus tendon express both tendon- and stem cell-related markers. Histochem. Cell Biol..

[35] Dex S., Lin D., Shukunami C., Docheva D. (2016). Tenogenic modulating insider factor: Systematic assessment on the functions of tenomodulin gene. Gene.

[36] Lovati A.B., Corradetti B., Lange Consiglio A., Recordati C., Bonacina E., Bizzaro D., Cremonesi F. (2011). Characterization and differentiation of equine tendon-derived progenitor cells. J. Biol. Regul. Homeost. Agents.

[37] Yang J., Zhao Q., Wang K., Liu H., Ma C., Huang H., Liu Y. (2016). Isolation and biological characterization of tendon-derived stem cells from fetal bovine. In Vitro Cell. Dev. Biol. Anim..

[38] Lee C.H., Lee F.Y., Tarafder S., Kao K., Jun Y., Yang G., Mao J.J. (2015). Harnessing endogenous stem/progenitor cells for tendon regeneration. J. Clin. Invest..

[39] Jo C.H., Kim O.S., Park E.Y., Kim B.J., Lee J.H., Kang S.B., Lee J.H., Han H.S., Rhee S.H., Yoon K.S. (2008). Fetal mesenchymal stem cells derived from human umbilical cord sustain primitive characteristics during extensive expansion. Cell Tissue Res..

[40] Staverosky J.A., Pryce B.A., Watson S.S., Schweitzer R. (2009). Tubulin polymerization-promoting protein family member 3, Tppp3, is a specific marker of the differentiating tendon sheath and synovial joints. Dev. Dyn..

[41] Harvey T., Flamenco S., Fan C.M. (2019). A *Tppp3^+^Pdgfra^+^* tendon stem cell population contributes to regeneration and reveals a shared role for PDGF signalling in regeneration and fibrosis. Nat. Cell Biol..

[42] Yin Z., Hu J.J., Yang L., Zheng Z.F., An C.R., Wu B.B., Zhang C., Shen W.L., Liu H.H., Chen J.L. (2016). Single-cell analysis reveals a nestin^+^ tendon stem/progenitor cell population with strong tenogenic potentiality. Sci. Adv..

[43] Lin D., Alberton P., Caceres M.D., Volkmer E., Schieker M., Docheva D. (2017). Tenomodulin is essential for prevention of adipocyte accumulation and fibrovascular scar formation during early tendon healing. Cell Death Dis..

[44] Takahashi K., Yamanaka S. (2006). Induction of pluripotent stem cells from mouse embryonic and adult fibroblast cultures by defined factors. Cell.

[45] Ohnishi K., Semi K., Yamamoto T., Shimizu M., Tanaka A., Mitsunaga K., Okita K., Osafune K., Arioka Y., Maeda T. (2014). Premature termination of reprogramming in vivo leads to cancer development through altered epigenetic regulation. Cell.

[46] Mosteiro L., Pantoja C., Alcazar N., Marion R.M., Chondronasiou D., Rovira M., Fernandez-Marcos P.J., Munoz-Martin M., Blanco-Aparicio C., Pastor J. (2016). Tissue damage and senescence provide critical signals for cellular reprogramming in vivo. Science.

[47] Papp B., Plath K. (2013). Epigenetics of reprogramming to induced pluripotency. Cell.

[48] Cevallos R.R., Edwards Y.J.K., Parant J.M., Yoder B.K., Hu K. (2020). Human transcription factors responsive to initial reprogramming predominantly undergo legitimate reprogramming during fibroblast conversion to iPSCs. Sci. Rep..

[49] Saitoh I., Sato M., Kiyokawa Y., Inada E., Iwase Y., Ibano N., Noguchi H. (2021). Induced Tissue-Specific Stem Cells (iTSCs): Their generation and possible use in regenerative medicine. Pharmaceutics.

[50] Sarkar T.J., Quarta M., Mukherjee S., Colville A., Paine P., Doan L., Tran C.M., Chu C.R., Horvath S., Qi L.S. (2020). Transient non-integrative expression of nuclear reprogramming factors promotes multifaceted amelioration of aging in human cells. Nat. Commun..

[51] Racila D., Winter M., Said M., Tomanek-Chalkley A., Wiechert S., Eckert R.L., Bickenbach J.R. (2011). Transient expression of OCT4 is sufficient to allow human keratinocytes to change their differentiation pathway. Gene Ther..

[52] Ring K.L., Tong L.M., Balestra M.E., Javier R., Andrews-Zwilling Y., Li G., Walker D., Zhang W.R., Kreitzer A.C., Huang Y. (2012). Direct reprogramming of mouse and human fibroblasts into multipotent neural stem cells with a single factor. Cell Stem Cell.

[53] Nemajerova A., Kim S.Y., Petrenko O., Moll U.M. (2012). Two-factor reprogramming of somatic cells to pluripotent stem cells reveals partial functional redundancy of Sox2 and Klf4. Cell Death Differ..

[54] Xu Y., Shi Y., Ding S. (2008). A chemical approach to stem-cell biology and regenerative medicine. Nature.

[55] Feng B., Ng J.H., Heng J.C., Ng H.H. (2009). Molecules that promote or enhance reprogramming of somatic cells to induced pluripotent stem cells. Cell Stem Cell.

[56] Li Y., Zhang Q., Yin X., Yang W., Du Y., Hou P., Ge J., Liu C., Zhang W., Zhang X. (2011). Generation of iPSCs from mouse fibroblasts with a single gene, Oct4, and small molecules. Cell Res..

[57] Hou P., Li Y., Zhang X., Liu C., Guan J., Li H., Zhao T., Ye J., Yang W., Liu K. (2013). Pluripotent stem cells induced from mouse somatic cells by small-molecule compounds. Science.

[58] Yang J.H., Petty C.A., Dixon-McDougall T., Lopez M.V., Tyshkovskiy A., Maybury-Lewis S., Tian X., Ibrahim N., Chen Z., Griffin P.T. (2023). Chemically induced reprogramming to reverse cellular aging. Aging.

[59] Li X., Liu D., Ma Y., Du X., Jing J., Wang L., Xie B., Sun D., Sun S., Jin X. (2017). Direct reprogramming of fibroblasts via a chemically induced XEN-like state. Cell Stem Cell.

[60] Shi Y., Do J.T., Desponts C., Hahm H.S., Scholer H.R., Ding S. (2008). A combined chemical and genetic approach for the generation of induced pluripotent stem cells. Cell Stem Cell.

[61] Shi Y., Desponts C., Do J.T., Hahm H.S., Scholer H.R., Ding S. (2008). Induction of pluripotent stem cells from mouse embryonic fibroblasts by Oct4 and Klf4 with small-molecule compounds. Cell Stem Cell.

[62] Mikkelsen T.S., Hanna J., Zhang X., Ku M., Wernig M., Schorderet P., Bernstein B.E., Jaenisch R., Lander E.S., Meissner A. (2008). Dissecting direct reprogramming through integrative genomic analysis. Nature.

[63] Huangfu D., Maehr R., Guo W., Eijkelenboom A., Snitow M., Chen A.E., Melton D.A. (2008). Induction of pluripotent stem cells by defined factors is greatly improved by small-molecule compounds. Nat. Biotechnol..

[64] Huangfu D., Osafune K., Maehr R., Guo W., Eijkelenboom A., Chen S., Muhlestein W., Melton D.A. (2008). Induction of pluripotent stem cells from primary human fibroblasts with only Oct4 and Sox2. Nat. Biotechnol..

[65] Silva J., Barrandon O., Nichols J., Kawaguchi J., Theunissen T.W., Smith A. (2008). Promotion of reprogramming to ground state pluripotency by signal inhibition. PLoS Biol..

[66] Li W., Wei W., Zhu S., Zhu J., Shi Y., Lin T., Hao E., Hayek A., Deng H., Ding S. (2009). Generation of rat and human induced pluripotent stem cells by combining genetic reprogramming and chemical inhibitors. Cell Stem Cell.

[67] Zhao Y., Zhao T., Guan J., Zhang X., Fu Y., Ye J., Zhu J., Meng G., Ge J., Yang S. (2015). A XEN-like state bridges somatic cells to pluripotency during chemical reprogramming. Cell.

[68] Guan J., Wang G., Wang J., Zhang Z., Fu Y., Cheng L., Meng G., Lyu Y., Zhu J., Li Y. (2022). Chemical reprogramming of human somatic cells to pluripotent stem cells. Nature.

[69] Zhang Y., Liu Y., Liu H., Tang W.H. (2019). Exosomes: Biogenesis, biologic function and clinical potential. Cell Biosci..

[70] Conboy I.M., Conboy M.J., Wagers A.J., Girma E.R., Weissman I.L., Rando T.A. (2005). Rejuvenation of aged progenitor cells by exposure to a young systemic environment. Nature.

[71] Liu S., Mahairaki V., Bai H., Ding Z., Li J., Witwer K.W., Cheng L. (2019). Highly purified human extracellular vesicles produced by stem cells alleviate aging cellular phenotypes of senescent human cells. Stem Cells.

[72] Zhang Y., Xu J., Liu S., Lim M., Zhao S., Cui K., Zhang K., Wang L., Ji Q., Han Z. (2019). Embryonic stem cell-derived extracellular vesicles enhance the therapeutic effect of mesenchymal stem cells. Theranostics.

[73] Yu H., Cheng J., Shi W., Ren B., Zhao F., Shi Y., Yang P., Duan X., Zhang J., Fu X. (2020). Bone marrow mesenchymal stem cell-derived exosomes promote tendon regeneration by facilitating the proliferation and migration of endogenous tendon stem/progenitor cells. Acta Biomater..

[74] Khanh V.C., Yamashita T., Ohneda K., Tokunaga C., Kato H., Osaka M., Hiramatsu Y., Ohneda O. (2020). Rejuvenation of mesenchymal stem cells by extracellular vesicles inhibits the elevation of reactive oxygen species. Sci. Rep..

[75] Zhang N., Zhu J., Ma Q., Zhao Y., Wang Y., Hu X., Chen J., Zhu W., Han Z., Yu H. (2020). Exosomes derived from human umbilical cord MSCs rejuvenate aged MSCs and enhance their functions for myocardial repair. Stem Cell Res. Ther..

[76] van der Windt D.A., Koes B.W., Boeke A.J., Deville W., De Jong B.A., Bouter L.M. (1996). Shoulder disorders in general practice: Prognostic indicators of outcome. Br. J. Gen. Pract..

[77] Yao X., Wei W., Wang X., Chenglin L., Bjorklund M., Ouyang H. (2019). Stem cell derived exosomes: microRNA therapy for age-related musculoskeletal disorders. Biomaterials.

[78] Yu H., Yuan Y., Shen H., Cheng T. (2006). Hematopoietic stem cell exhaustion impacted by p18 INK4C and p21 Cip1/Waf1 in opposite manners. Blood.

[79] Singh S., Jakubison B., Keller J.R. (2020). Protection of hematopoietic stem cells from stress-induced exhaustion and aging. Curr. Opin. Hematol..

[80] Hashimoto M., Kamphorst A.O., Im S.J., Kissick H.T., Pillai R.N., Ramalingam S.S., Araki K., Ahmed R. (2018). CD8 T cell exhaustion in chronic infection and cancer: Opportunities for interventions. Annu. Rev. Med..

[81] Monsel A., Zhu Y.G., Gennai S., Hao Q., Liu J., Lee J.W. (2014). Cell-based therapy for acute organ injury: Preclinical evidence and ongoing clinical trials using mesenchymal stem cells. Anesthesiology.

[82] Revuelta M., Matheu A. (2017). Autophagy in stem cell aging. Aging Cell.

[83] Heinemeier K.M., Schjerling P., Heinemeier J., Magnusson S.P., Kjaer M. (2013). Lack of tissue renewal in human adult Achilles tendon is revealed by nuclear bomb ^14^C. FASEB J..

[84] Heinemeier K.M., Schjerling P., Ohlenschlaeger T.F., Eismark C., Olsen J., Kjaer M. (2018). Carbon-14 bomb pulse dating shows that tendinopathy is preceded by years of abnormally high collagen turnover. FASEB J..

[85] Stolzing A., Jones E., McGonagle D., Scutt A. (2008). Age-related changes in human bone marrow-derived mesenchymal stem cells: Consequences for cell therapies. Mech. Ageing Dev..

[86] Weiss C., Kornicka-Grabowska K., Mularczyk M., Siwinska N., Marycz K. (2020). Extracellular microvesicles (MV’s) isolated from 5-azacytidine-and-resveratrol-treated cells improve viability and ameliorate endoplasmic reticulum stress in metabolic syndrome derived mesenchymal stem cells. Stem Cell Rev. Rep..

